# Determining hematological, biochemical and immunological reference values in healthy adults with high-risk for HIV acquisition in Mozambique

**DOI:** 10.1371/journal.pone.0232018

**Published:** 2020-04-30

**Authors:** Victória Cumbane, Michelle Imbach, Raquel Matavele Chissumba, Ivalda Macicame, Leigh Anne Eller, John Lawlor, Mark Milazzo, Qun Li, Trevor Crowell, Mirna Mutombene, Onélia Guiliche, Edna Viegas, Chiaka Nwoga, Adam Yates, Nelson Michael, Merlin Robb, Christina S. Polyak, Ilesh V. Jani, Nilesh Bhatt

**Affiliations:** 1 Centro de Investigação e Treino em Saúde da Polana Caniço (CISPOC), Instituto Nacional de Saúde (INS), Ministério da Saúde (MISAU), Maputo, Mozambique; 2 Military HIV Research Program, Walter Reed Army Institute of Research, Silver Spring, Maryland, United States of America; 3 Henry Jackson Foundation for the Advancement of Military Medicine, Bethesda, Maryland, United States of America; Oregon State University, UNITED STATES

## Abstract

**Introduction:**

In many African countries, laboratory reference values are not established for the local healthy adult population. In Mozambique, reference values are known for young adults (18-24yo) but not yet established for a wider age range. Our study aimed to establish hematological, biochemical and immunological reference values for vaccine trials in Mozambican healthy adults with high-risk for HIV acquisition.

**Methods:**

A longitudinal cohort and site development study in Mozambique between November 2013 and 2014 enrolled 505 participants between 18 to 35 years old. Samples from these healthy participants, were analyzed to determine reference values. All volunteers included in the analysis were clinically healthy and human immunodeficiency virus (HIV), hepatitis B and C virus, and syphilis negative. Median and reference ranges were calculated for the hematological, biochemical and immunological parameters. Ranges were compared with other African countries, the USA and the US National Institute of Health (NIH) Division of AIDS (DAIDS) toxicity tables.

**Results:**

A total of 505 participant samples were analyzed. Of these, 419 participants were HIV, hepatitis B and C virus and syphilis negative including 203 (48.5%) females and 216 (51.5%) males, with a mean age of 21 years. In the hematological parameters, we found significant differences between sex for erythrocytes, hemoglobin, hematocrit, MCV, MCH and MCHC as well as white blood cells, neutrophils and platelets: males had higher values than females. There were also significant differences in CD4^+^T cell values, 803 cells/μL in men versus 926 cells/μL in women. In biochemical parameters, men presented higher values than women for the metabolic, enzymatic and renal parameters: total and direct bilirubin, ALT and creatinine.

**Conclusion:**

This study has established reference values for healthy adults with high-risk for HIV acquisition in Mozambique. These data are helpful in the context of future clinical research and patient care and treatment for the general adult population in the Mozambique and underline the importance of region-specific clinical reference ranges.

## Introduction

In Mozambique, the HIV epidemic is continuing to grow, with 223 new infections every day and a national prevalence of 13.2% [1]. HIV prevalence is higher among women (15.4%) than men (10.1%) and in those aged 15 to 49 years, peaking at 35 to 39 years old [2]. Due to the high prevalence of HIV, numerous clinical trials ranging from Phase I safety studies to Phase III HIV vaccine efficacy studies are ongoing in Mozambique. These studies require clinical laboratory reference values to allow for accurate screening and enrollment of volunteers.

Laboratory reference values are needed to evaluate worsening pre-existing conditions, the occurrence of new conditions, adverse vaccine reactions and toxicity related to investigational products in volunteers participating in clinical trials. A prior study conducted in Mozambique attempted to establish normal reference values, however, it lacked the recommended age and gender diversity per national laboratory guidelines since it focused on young adults, aged 18–24 years old [3]. Therefore, laboratory reference values have not yet been obtained for the local healthy adult population in Mozambique.

Given the limited data, many health care providers and researchers are using reference values for clinical laboratory parameters provided by the equipment manufacturers and/or described in textbooks, which are typically based on data collected from European and North American populations. Several studies have shown that reference values arising from individuals living in North America and Europe are significantly different than those from individuals living in Africa [4–9]. In the context of clinical studies, these differences in laboratory ranges may cause inappropriate exclusion of potential participants and may lead to erroneous classification of adverse events.

As part of the effort to establish clinical and laboratory capacity to conduct HIV vaccine trials in Mozambique, a study was conducted to assess the incidence of HIV infection, retention rate and willingness of adults to participate in future HIV vaccine trials in Mozambique (RV363). As part of this study, hematological, biochemical and immunological reference values for healthy adults in Mozambique were determined.

## Materials and methods

### Study population

The enrolled participants were part of a cohort and site development study that evaluated incidence of HIV infection, retention rate and willingness of adults to participate in future HIV Vaccine Trials in Mozambique (RV363). Briefly, 505 male and female participants aged 18 to 35 years with high-risk for HIV acquisition (2 or more sexual partners in last 3 months) were enrolled in the parent study between November 2013 and November 2014. Samples, described below, for reference values were collected upon study start. Participants who were HIV negative, syphilis, Hepatitis B and C negative, and malaria negative, as per below, and women who were not pregnant were included in this analysis. Samples were also excluded from this analysis if results for lymphocyte immunophenotyping, clinical chemistry, and/ or hematology were missing.

The study was conducted at the Polana Caniço Health Research and Training Center (CISPOC), a research center of the Instituto Nacional de Saúde (INS), Ministry of Health in Maputo, Mozambique. CISPOC is located in the neighborhood of a periurban area of Polana Caniço of 115,000 residents. This area is characterized by high population density and poverty, and the main source of income is informal trade. In Maputo city, the HIV prevalence is 16.9%, and higher in women (21.7%) than in men (11.0%) [2].

The study was approved by the National Bioethics Committee for Health (CNBS), Ministry of Health (MISAU) from Mozambique (reference letter 282/CNBS/13 dated October 2^nd^ 2013) and Walter Reed Army Institute of Research from the USA (approval letter dated October 25^th^ 2013, US Army Medical Research and Material Command of -2-0174 the United States in Fort Detrick, Maryland–Award number W81WH-11-2-0174). Written informed consent was obtained from each participant prior of conducting any study procedures.

### Laboratory testing

All blood samples were collected and analyzed at the CISPOC laboratory to determine the hematological, biochemical and immunological parameters. Parent study participants with evidence of malaria, pregnancy, syphilis, hepatitis, and HIV were excluded from this analysis. All laboratory testing was performed according to the manufacturer's instructions.

#### HIV, syphilis, malaria and hepatitis testing

The Mozambican national algorithm was used for diagnosis of HIV, composed of two sequential immunochromatographic rapid tests, the Determine^TM^ HIV-1/2 (Alere Medical CO., Ltd Japan) for screening, followed by the UniGold^®^ HIV-1/2 (Trinity Biotech, Bray, Ireland), for confirmation of positive results. In case of discordant HIV results, a fourth-generation test was performed by using an enzyme immunoassay ELISA (Genscreen^™^ ULTRA HIV Ag-Ab; Bio-Rad, France). Syphilis testing was performed by using non-treponemal test card, RPR (Macro-Vue RPR Card Tests, BD, Ireland) for screening and confirmed by a treponemal test TPPA (Serodia TP-PA, Fujirebio INC, Japan). Hepatitis B surface antigen (HBsAg) testing was preformed using an enzyme immunoassay EIA (Genscreen^TM^ HBsAg EIA 3.0). Screening rapid tests were performed using the OraQuick HCV Rapid antibody test (OraSure Technologies, Inc. Bethlehem, PA USA), and confirmation for anti-Hepatitis C was performed using an anti-HCV ELISA (Ortho(R) HCV Version 3.0 ELISA Test System). Malaria testing was performed by using an immunochromatographic test (Clearview Malaria, Inverness Medical, South Africa).

#### Hematologic and biochemical testing

Full blood count including three-part differential of hematological parameters was performed within 6 hours of blood collection in Vacutainer^®^ tubes with K2(EDTA) anticoagulant (Becton Dickinson, USA) using the hematology analyzer Sysmex KX21N (Sysmex Corporation, Japan). For the biochemical parameters (total bilirubin, direct bilirubin, alanine aminotransferase (ALT), creatinine and glucose), the samples were collected in tubes without anticoagulant and centrifuged at 2500 rpm for 15 minutes. Serum aliquots were processed in the chemistry analyzer Vitalab Selectra Junior^®^ (Vital Scientific, Netherlands) within 24 hours after collection.

#### Immunophenotyping analysis by flow cytometry

The immunophenotyping parameters were determined in TruCOUNT tube BD (Becton Dickinson, USA) in which 20μl of a cocktail of monoclonal antibodies (CD3^FITC^+/CD8^PE^+/CD45+^PerCP^/CD4^APC^—Becton Dickinson, USA) were mixed in 50 μl of whole blood cell and incubated in the dark at room temperature for 15 minutes. The red blood cells were lysed by adding 450 μl of a lysing solution (FACS Lysing—Becton Dickinson, USA) incubated in the dark, at room temperature for another 15 minutes. At the end, the samples were read on FACS Calibur flow cytometer (Becton Dickinson, USA) using the Multiset software (Becton Dickinson, USA) within a period of 24 hours after sample preparation.

#### Pregnancy test

Urine pregnancy test was performed using the hCG combo cassette (Alere, Australia).

#### Quality control

Daily internal quality controls were run in hematology, biochemistry and immunophenotyping analyzers following the manufacturer instructions. In addition to the internal controls, the CISPOC laboratory participates in external proficiency testing three times a year distributed over the College of American Pathologists, USA (for hematology and biochemistry) and the Oneworld Accuracy, Canada, (for HIV, syphilis serology and pregnancy test). For CD4 testing, the laboratory participates in the International Quality Assurance Program-QASI from the Public Health Agency of Canada, Ottawa. The clinical and laboratory staff were trained in Good Clinical Practice and Good Clinical and Laboratory Practice, respectively.

### Adverse events

The 2014 Division of AIDS (DAIDS Table for Grading the Severity of Adult and Pediatric Adverse Events) was used to describe the frequency of potential adverse events and to select eligible participants for future HIV prevention studies from our reference values [10].

### Statistical analysis

Conforming to established practices in the literature regarding the formation of normal lab values [11], we first exclude unusual values (“outliers”) defined by Tukey as measurements beyond the 75^th^ and 25^th^ percentile +/- 1.5 x Inter-Quartile Range (IQR). We explored using wider ranges at +/- 2.0/2.5/3.0 x IQR to eliminate fewer outliers. Clinical authors felt there was little practical difference between using these higher Tukey “fence” cutoff multipliers 2.0/2.5/3.0, therefore adhere to the standard 75^th^/25^th^ percentile +/- 1.5 x IQR as the outlier cutoff/elimination point and present the middle 95%: 2.5^th^ percentile to 97.5^th^ percentile of those remaining.

We then compared by sex using the two-tailed Wilcoxon rank sum test. The Wilcoxon rank sum test is a valid approach given the non-normality of most of the lab tests and the reliance on convenience sampling in data collection. However, while the Wilcoxon test is robust against assumptions of normality, as a validation of the data’s internal validity we also chose to normalize the data using the Box-Cox transformation method [12] in SAS version 9.4 using the TRANSREG Procedure. The transformed values where then compared by sex using the t-test. In all cases, the Wilcoxon rank sum test and the t-test on the normalized data returned the same results. Thus, we present the Wilcoxon rank sum results only. The clinical chemistry, hematologic, and immunophenotyping values from this study were presented alongside data from participants at low risk for HIV in Mozambique, from South African (as per the BARC Clinical Trials Laboratory), as well as the United States American Board of Internal Medicine [13,14].

## Results

Among the 505 HIV negative participants, 86 were excluded due to following reasons: malaria (n = 1), missing CBC values (n = 16), positive syphilis test (n = 15), pregnancy (n = 7), Hepatitis B (n = 47), Hepatitis C (n = 1), and seroconversion to HIV-1 (n = 3). One seroconverter was also pregnant. Of those testing positive for Hepatitis B surface antigen (HBsAg), one participant was also syphilis positive, one pregnant and one had missing CBC values. Therefore, a total of 419 healthy volunteer samples were available for analysis of the reference values comprising 216 males (51.5%) and 203 females (48.5%) with a median (IQR) age of 21 years (19, 24).

### Hematology

Hematology reference values were established for 17 parameters. Table 1 shows the reference values (median and 2.5th—97.5^th^ percentiles) for these parameters. Statistically significant differences (p <0.05) between sex were observed in all parameters, except mean platelet volume (MPV), lymphocyte percentage and neutrophil percentage. Table 2 shows hematology comparisons with other local and international reference values.

**Table 1:** Hematology reference values (median and 2.5^th^- 97.5^th^ percentiles) derived from healthy adults at high-risk for HIV in Mozambique.

Parameter	Total		Male		Female		*P*-value
	n	Range	n	Range	n	Range	
Hemoglobin (g/dL)	410	13.3 (8.9–16.4)	211	14.6 (12.6–16.9)	199	12.0 (7.7–14.3)	<0.0001
Hematocrit (%)	410	39.9 (24.6–48.8)	211	43.2 (33.5–49.8)	199	35.8 (22.1–43.8)	<0.0001
Erythrocytes (10^6^/μL)	410	4.8 (3.1–6.1)	211	5.1 (3.9–6.3)	199	4.4 (2.7–5.5)	<0.0001
Platelets (10^3^/μL)	410	249.0 (141–439)	211	227 (129–350)	199	280 (157–533)	<0.0001
WBC (10^3^/μL)	410	4.8 (2.9–8.3)	211	4.5 (2.8–7.8)	199	5.1 (3.2–8.7)	<0.0001
MCH (pg)	410	27.9 (19.8–44.0)	211	28.8 (22.5–33.8)	199	26.7 (18.5–45.6)	<0.0001
MCV (fl)	410	83.2 (66.0–93.8)	211	85.0 (71.1–94.2)	199	80.5 (63.8–92.9)	<0.0001
MCHC (g/L)	410	33.4 (28.8–54.8)	211	33.9 (29.4–37.1)	199	32.9 (28.0–55.2)	<0.0001
Neutrophils (10^3^/μL)	410	2.7 (1.2–5.8)	211	2.5 (1.1–5.3)	199	2.8 (1.4–6.0)	<0.0001
Neutrophils (%)	410	56.2 (36.3–73.6)	211	54.7 (35.7–73.3)	199	57.2 (38.6–74.6)	0.0716
Lymphocytes (10^3^/μL)	410	1.8 (1.0–2.9)	211	1.7 (1.0–2.7)	199	1.9 (1.3–3.3)	0.0077
Lymphocytes (%)	410	37.4 (21.0–55.2)	211	38.3 (21.8–55.2)	199	36.5 (20.8–55.4)	0.0773
MXD (10^3^/μL)	407	0.3 (0.0–0.7)	209	0.3 (0.0–0.7)	198	0.3 (0.1–0.8)	0.1899
MXD (%)	408	6.5 (0.8–13.6)	209	6.7 (0.9–14.0)	199	6.3 (0.7–13.6)	0.2126
MPV (fL)	405	9.5 (7.9–11.6)	209	9.4 (8.0–11.3)	196	9.5 (7.7–11.8)	0.7507
RDW_CV (%)	409	14.2 (12.1–20.1)	210	13.9 (12.1–18.1)	199	14.5 (12.3–21.3)	0.0485
RDW_SD (fL)	408	43.8 (38.6–52.2)	210	43.2 (38.1–50.5)	198	44.5 (38.9–52.7)	0.0008

*p*-values indicate comparisons between males and females using Wilcoxon rank-sum test. WBC: white blood cell; MCH: Mean corpuscular hemoglobin; MCV: Mean corpuscular volume; MCHC: Mean corpuscular hemoglobin concentration; MXD: Mixed cell percent; MPV: Mean platelet volume; RDW_CV: Red cell distribution width as coefficient of variation; RDW_SD: Red cell distribution width as standard deviation

**Table 2:** Hematology reference ranges from healthy adults at high-risk for HIV in Mozambique compared with local (low risk healthy adults), regional (south africa) and international countries (USA).

Parameter	High-risk healthy adults in Maputo (18–35 years old)	Low risk young adults in Maputo (18–24 years old)	BARC SA Clinical Trials Laboratory (13–108 years old)	USA
**Hemoglobin (g/dL)**				
Male	12.6–16.9	12.3–16.4	13.8–18.8	14.0–18.0
Female	7.7–14.3	7.0–13.1	12.4–16.7	12.0–16.0
All	8.9–16.4	7.5–15.8	-	-
**Hematocrit (%)**				
Male	33.5–49.8	25.2–50.4	40.0–56.0	42.0–50.0
Female	22.1–43.8	19.5–40.3	35.0–49.0	37.0–47.0
All	24.6–48.8	202.0–47.2		
**Erythrocytes (10**^**6**^**/**μ**L)**				
Male	3.9–6.3	2.7–6.1	4.5–6.5	-
Female	2.7–5.5	2.3–5.0	3.8–5.5	-
All	3.1–6.1	2.4–5.9	-	4.2–5.9
**Platelets (10**^**3**^**/**μ**L)**				
Male	129–350	116.2–392.1	-	-
Female	157–533	128.8–503.0	-	-
All	141–439	125.2–488.0	150.0–450.0	150.0–450.0
**WBC (10**^**3**^**/**μ**L)**				
Male	2.8–7.8	2.9–7.7	-	-
Female	3.2–8.7	3.2–9.1	-	-
All	2.9–8.3	3.0–8.7	4.0–12.0	4.0–11.0
**Neutrophils (10**^**3**^**/**μ**L)**			-	-
Male	1.1–5.3	1.1–5.1	-	-
Female	1.4–6.0	1.4–7.0	-	
All	1.2–5.8	1.2–6.1	2.0–7.5	2.0–8.3
**Neutrophils (%)**			-	-
Male	35.7–73.0	34.4–70.8	-	-
Female	38.6–74.6	37.0–76.7	-	-
All	36.3–73.6	34.9–74.9	-	50.0–70.0
**Lymphocytes (10**^**3**^**/**μ**L)**			-	
Male	1.0–2.7	1.1–3.3	-	-
Female	1.3–3.3	1.0–3.1	-	-
All	1.0–2.9	1.1–3.1	1.0–4.0	-
**Lymphocytes (%)**			-	-
Male	21.8–55.2	15.5–57.1	-	-
Female	20.8–55.4	17.8–53.6	-	-
All	210–55.2	16.6–56.2	-	30.0–45.0
**MCHC (g/L)**				-
Male	29.4–37.1	30.4–54.2	-	-
Female	28.0–55.2	25.8–56.1	-	-
All	28.-54.8	28.1–55.9	32.0–36.0	33.0–36.0
**RDW-CV (%)**			-	-
Male	12.1–18.1	11.6–18.6	-	-
Female	12.3–21.3	12.2–23.5	-	-
All	12.1–20.1	11.9–23.4	-	9.0–14.5
**RDW-SD (fl)**			-	-
Male	38.1–50.5	37.0–50.0	-	-
Female	38.9–52.7	36.3–52.6	-	-
All	386–52.2	36.4–52.2	-	-
**MPV (fL)**			-	-
Male	8.0–11.3	8.7–13.1	-	-
Female	7.7–11.8	8.5–12.7	-	-
All	7.9–11.6	8.5–13.0	-	-

The comparison values represent established ranges from sources [3,13,14]

### Immunophenotyping

Immunophenotyping reference values were established for three parameters. Table 3 presents the reference values (median and 2.5^th^– 97.5% percentiles) for these parameters. The CD3 absolute count, CD4 absolute count, and CD4 percentage were statistically significantly higher (p<0.05) for females compared to men using the Wilcoxon rank-sum test. Table 4 shows immunophenotyping comparisons with other local and international reference values.

**Table 3:** Lymphocytes subset reference values (median and 2.5^th^—97.5^th^ percentiles) derived from healthy adults at high-risk for HIV in Mozambique.

Parameter	Total		Male		Female		*P*-values
	n	Range	n	Range	n	Range	
CD3+ cells/uL	392	1450 (861–2571)	199	1370 (839–2298)	193	1551 (935–2675)	0.0004
CD3+ T cells/uL (%)	392	70.0 (54.0–81.0)	199	69.0 (49.0–82.0)	193	71.0 (56.0–81.0)	0.2399
CD4+ T cells/uL	414	856 (455–1536)	214	803 (433–1283)	200	926 (531–1616)	<0.0001
CD4+ T cells/uL (%)	414	41.0 (29.0–53.0)	214	39.5 (28.0–53.0)	200	42.0 (32.0–53.0)	0.0001
CD8+ T cells/uL	392	500 (235–958)	199	483 (227–974)	193	525 (260–957)	0.1304
CD8+ T cells/uL (%)	392	24.0 (14.0–36.0)	199	24.0 (13.0–37.0)	193	24.0 (14.0–33.0)	0.1650

*p*-values indicate comparisons between males and females using the Wilcoxon rank-sum test.

**Table 4:** Lymphocytes subset reference values from healthy adults at high-risk for HIV in Mozambique compared with local (low risk healthy adults), regional (SA) and international countries (USA).

Parameter	Healthy adults in Maputo (18–35 years old)	Low risk young adults in Maputo (18–24 years old)	BARC SA Clinical Trials Laboratory (13–108 years old)	USA
**CD3+T cells/μL**				
Male	839–2298	716–1917	-	-
Female	935–2675	756–2313	-	-
All	861–2571	729–2220	723–2737	900–3245
**CD3+T cells/uL (%)**				
Male	49.0–82.0	50.2–79.5	-	-
Female	56.0–81.0	55.1–80.9	-	-
All	54.0–81.0	51.7–80.7	56.0–86.0	-
**CD4+T cells/μL**				
Male	433–1283	357–1155	-	-
Female	531–1616	434–1479	-	-
All	455–1536	381–1340	500–2010	530–1570
**CD4+T cells/μL (%)**				
Male	28.0–53.0	23.2–49.1	-	-
Female	32.0–53.0	29.9–53.7	-	-
All	29.0–53.0	25.8–52.2	28.0–58.0	-
**CD8+T cells/μL**				
Male	227–974	214–902	-	-
Female	260–957	234–965	-	-
All	235–958	218–952	220–1129	430–1060
**CD8+T cells/μL (%)**				
Male	13.0–37.0	14.9–41.5	-	-
Female	14.0–33.0	14.7–34.5	-	-
All	14.0–36.0	14.7–37.6	13.0–39.0	-

The comparison values represent established ranges from sources [3,13,14]

### Chemistry

Chemistry reference values were established for five parameters: Alanine Aminotransferase (ALT), Creatinine, Total Bilirubin, Direct Bilirubin and Glucose. Table 5 presents the reference values (median and 2.5^th^– 97.5% percentiles) for these parameters. Statistically significant differences (p<0.0001) between sex using the Wilcoxon rank-sum test were observed for Alanine Aminotransferase, Creatinine, Total Bilirubin, and Direct Bilirubin, with males presenting higher values than females. Table 6 shows chemistry comparisons with other local and international reference values.

**Table 5:** Clinical chemistry reference ranges (median and 2.5^th^- 97.5^th^ percentiles) derived from healthy adults at high-risk for HIV in Mozambique.

Parameter	Total		Male		Female		*P*-values
	n	Range	n	Range	n	Range	
Total Bilirubin (μmol/L)	410	10.5 (4.6–33.7)	210	12.9 (5.3–38.7)	200	8.3 (3.9–20.3)	<0.0001
Direct Bilirubin (μmol/L)	411	6.0 (2.5–18.3)	211	7.8 (3.0–20.4)	200	4.7 (2.1–12.0)	<0.0001
Glucose (mmol/L)	403	4.6 (3.3–6.3)	206	4.7 (3.4–6.3)	197	4.6 (3.3–6.6)	0.4003
ALT (U/L)	410	15.0 (4.0–55.0)	210	19.0 (6.0–58.0)	200	12.0 (3.5–41.5)	<0.0001
Creatinine (mmol/L)	407	57.5 (34.5–87.5)	208	68.1 (41.6–91.1)	199	50.4 (33.6–79.6)	<0.0001

*p*-values indicate comparisons between males and females using the Wilcoxon rank-sum test.

ALT: Alanine aminotransferase

**Table 6:** Chemistry reference ranges derived from healthy adults at high-risk for HIV in Mozambique compared with local (low risk healthy adults), regional (SA) and international countries (USA).

Parameter	High-risk healthy adults in Maputo (18–35 years old)	Low risk young adults in Maputo (18–24 years old)	BARC SA Clinical Trials Laboratory (13–108 years old)	USA
**Total Bilirubin (μmol/L)**				
Male	5.3–38.7	5.8–36.0	-	-
Female	3.9–20.3	4.0–22.5	-	-
All	4.6–33.7	4.4–27.9	2.0–21.0	5.1–17.1
**Direct Bilirubin (μmol/L)**				
Male	3.0–20.4	-	-	-
Female	2.1–12.0	-	-	-
All	2.5–18.3	-	<3.4	1.7–5.13
**Glucose (μmol/L)**				
Male	3.4–6.3	3.1–5.7	-	-
Female	3.3–6.6	3.2–5.3	-	-
All	3.3–6.3	3.1–5.5	3.3–7.8	-
**ALT (U/L)**				
Male	6.0–58.0	6.5–53.2	10.0–40.0	-
Female	3.5–41.5	4.8–38.5	7.0–35.0	-
All	4.0–55.0	5.0–48.2	-	10.0–40.0
**Creatinine (mg/dL)**				
Male	41.6–91.1	58.2–109.0	62.0–106.0	-
Female	33.6–79.6	45.0–86.6	44.0–80.0	-
All	34.5–87.5	47.1–103.2	-	61.8–132.6

The comparison values represent established ranges from sources [3,13,14]

### Implications of reference values in clinical trials

We evaluated the frequency of study participants who met criteria at enrollment for potential adverse events using the 2014 NIH US DAIDS toxicity classification table [10]. Low levels of hemoglobin were seen only in women in 10 (5.0%) cases with grade 1, 11 (5.5%) cases with grade 2, 7 (3.5%) cases with grade 3, and 1 (0.5%) case with grade 4. Low CD4 level resulted in adverse events in 2 (0.5%) cases, both grade 1. For biochemistry parameters, we found high/low glucose levels in 10 (2.5%) cases with grade 1, low/high ALT levels in 7 (1.7%) cases with grade 1 and 1 (0.2%) cases with grade 2, low/high Total Bilirubin in 6 (1.5%) cases with grade 1, 1 (0.2%) case with grade 2 and 1 (0.2%) with grade 3, and high/low level of creatinine in 2 (0.5%) cases with grade 1 as described in Table 7.

**Table 7:** Frequency of predicted adverse events from healthy adults at high-risk for HIV in Mozambique in comparison with values from DAIDS toxicity grading tables.

Division of AIDS (DAIDS) toxicity grading									
Parameters		Grade 1		Grade 2		Grade 3		Grade 4	
	N	n	%	n	%	n	%	n	%
T CD4 (cells/ul)	414	2	0.5	0	0.0	0	0.0	0	0.0
Hemoglobin (g/dL), male	211	0	0.0	0	0.0	0	0.0	0	0.0
Hemoglobin (g/dL), female	199	10	5.0	11	5.5	7	3.5	1	0.5
WBC (10^3^/μL)	410	2	0.5	0	0.0	0	0.0	0	0.0
Platelets (10^3^/μL)	410	5	1.0	1	0.2	0	0.0	0	0.0
Neutrophils (10^3^/μL)	410	0	0.0	0	0.0	0	0.0	0	0.0
Glucose (mmol/L)	403	10	2.5	0	0.0	0	0.0	0	0.0
ALT (U/L)	410	7	1.7	1	0.2	0	0.0	0	0.0
Total Bilirubin (μmol/L)	410	6	1.5	1	0.2	1	0.2	0	0.0
Creatinine (mmol/L)	407	2	0.5	0	0.0	0	0.0	0	0.0

## Discussion

This study establishes references ranges for hematological, biochemical and immunological parameters in healthy Mozambican adults with high-risk for HIV acquisition. Multiple hematology parameters were tested in this study and the majority of the parameters were significantly different between males and females. Higher values were observed for erythrocytes, hemoglobin, hematocrit, MCV, MCH, and MCHC in males than in females as well as neutrophils, WBCs and platelets. Similar results were reported in the study conducted in Mozambican healthy young adults with low risk for HIV acquisition [3], and consistent with other previous reports [3,5,7,15–19]. However, these differences were not clinically relevant. The reason for higher values for hemoglobin, hematocrit and erythrocytes in males than females, have been attributed to influence of the androgen hormone on erythropoiesis in males and menstrual blood loss in females [8]. Like studies in other African countries such as Uganda [15], Ethiopia [19,20], Western Kenya [5,9] our study also showed that females had higher values of total WBC count and platelet than males.

We observed that the women had lower hemoglobin than the range derived from the US and South African (12.0 g/dL and 12.4 g/dL, respectively). It could be due to differences in genetic background between the Mozambican population and the South African and North American populations or due to other factors such as poor nutritional status, genetic red blood cell disorders or parasitic infections, which have been shown to account for low hemoglobin values [6,16].

In the immunophenotyping data, we found significant higher counts in females compared to males for total CD3+ absolute T cell counts and for CD4+ absolute counts and CD4+ percentages. The differences in sex for CD4 and CD3 counts are in agreement with previous reports [3,5,7–9,19,20]. The reasons for this inequality can be due the action of sex hormones at the immune system [21]. Some studies have proved that the human immune system suffers from a sexual dysmorphism, displaying different responses depending on the sex [21]. Studies indicate that females have a higher CD4:CD8 ratio due to the presence of a greater absolute number of CD4 lymphocytes and that hormones like estrogens have a great impact in promoting the increase of the CD4+T cells [21,22]. Overall, the reference values reported in the present study for lymphocyte subsets were comparable to ranges reported in young adults in Mozambique [3], as well as Western Kenya [9], Uganda [15], Southern Tanzania [19], South African adults [13] and North American populations [14].

Similar to hematological and immunological parameters, the reference values for most of biochemistry parameters showed statistically significant differences between sexes. Males had higher values than females for both metabolic parameters (Total Bilirubin and Direct Bilirubin) as well as for the enzymatic parameter (ALT) and for the kidney function parameter (Creatinine). We had observed the same findings in our previous study conducted in young adults with low risk for HIV acquisition [3] as well as in other African countries Western Kenya [6], Ugandan [15], Botswana [23]. However, when we compared the biochemistry parameters from our population with the South African population, the creatinine levels in males from our study were notably lower (41.6–91.1μmol/L) than the South African population (62–106 μmol/L).

The NIH US Division of AIDS (DAIDS) toxicity table for grading adverse events has been used in many clinical trials to select potential participants and to monitor treatment outcomes [10]. A large proportion of our study participants would have been excluded based on hemoglobin levels found at enrollment. In addition, the laboratory parameters are different between men and women in the NIH US DAIDS toxicity table. In our study, the majority of women would have been excluded due to hemoglobin and laboratory parameters. Similar data were seen in other studies included in the previous study of young adults conducted in Mozambique [3,9].

The present study had some limitations that may have influenced the laboratory reference value findings. We did not screen for parasitic infections and dietary components that may influence for some of the hematology and immunological parameters. Malaria tests were not performed routinely to screen asymptomatic cases. However, the ranges defined in this study complement those already established for young adults in Mozambique and may be suitable to use in the general adult population and, of course, for vaccine trials.
